# Perspective influences eye movements during real-life conversation: Mentalising about self versus others in autism

**DOI:** 10.1177/1362361320936820

**Published:** 2020-07-09

**Authors:** Mahsa Barzy, Heather J Ferguson, David M Williams

**Affiliations:** University of Kent, UK

**Keywords:** Autism, eye-tracking, perspective taking, real-life social interactions, topic of conversation

## Abstract

Social-communication is profoundly impaired among autistic individuals. Difficulties representing others’ mental states have been linked to modulations of gaze and speech, which have also been shown to be impaired in autism. Despite these observed impairments in ‘real-world’ communicative settings, research has mostly focused on lab-based experiments, where the language is highly structured. In a pre-registered experiment, we recorded eye movements and verbal responses while adults (*N* = 50) engaged in a real-life conversation. Using a novel approach, we also manipulated the perspective that participants adopted by asking them questions that were related to the self, a familiar other, or an unfamiliar other. Results replicated previous work, showing reduced attention to socially relevant information among autistic participants (i.e. less time looking at the experimenter’s face and more time looking around the background), compared to typically developing controls. Importantly, perspective modulated social attention in both groups; talking about an unfamiliar other reduced attention to potentially distracting or resource-demanding social information and increased looks to non-social background. Social attention did not differ between self and familiar other contexts, reflecting greater shared knowledge for familiar/similar others. Autistic participants spent more time looking at the background when talking about an unfamiliar other versus themselves. Future research should investigate the developmental trajectory of this effect and the cognitive mechanisms underlying it.

**Lay abstract:**

Previous lab-based studies suggest that autistic individuals are less attentive to social aspects of their environment. In our study, we recorded the eye movements of autistic and typically developing adults while they engaged in a real-life social interaction with a partner. Results showed that autistic adults were less likely than typically developing adults to look at the experimenter’s face, and instead were more likely to look at the background. Moreover, the perspective that was adopted in the conversation (talking about self versus others) modulated the patterns of eye movements in autistic and non-autistic adults. Overall, people spent less time looking at their conversation partner’s eyes and face and more time looking at the background, when talking about an unfamiliar other compared to when talking about themselves. This pattern was magnified among autistic adults. We conclude that allocating attention to social information during conversation is cognitively effortful, but this can be mitigated when talking about a topic that is familiar to them.

Autism spectrum disorder (ASD) is a pervasive neurodevelopmental condition, diagnosed on the basis of impairments in social-communication and a restricted and repetitive pattern of behaviour and interests (American Psychiatric Association, 2013). Two key cognitive-level mechanisms that underpin social-communication ability are theory of mind (ToM) and social attention (Chevallier et al., 2012; Kalandadze et al., 2018). ToM is the ability to represent the mental states of the self and others in order to explain and predict behaviour and is widely believed to be impaired among autistic people^1^ (Baron-Cohen et al., 1985; Happé, 1994; Moran et al., 2011; Premack & Woodruff, 1978). Social attention, which refers to the ability and motivation to attend to, as well as coordinate attention with, a social partner during interaction (e.g. through joint attention, use of non-verbal gestures, including eye contact and orientation and focusing of the visual system towards one’s partner), is also known to be atypical in autism (Chita-Tegmark, 2016). Social attention and ToM can be modulated through eye gaze, because we send and receive a great deal of social information through use and shifting of gaze (Cañigueral & de C. Hamilton, 2019). It is particularly notable, therefore, that social-communication and ToM impairments in autism are associated with an atypical social attention distribution (Senju, 2013; Swettenham et al., 1998; von dem Hagen et al., 2013).

Previous studies have adopted a range of tasks and stimuli (e.g. images of isolated faces, static cartoon/natural images, dynamic videos etc) to examine how autism impacts social attention (Bhat et al., 2010; Bird et al., 2011; Chawarska et al., 2013; Corden et al., 2008; Dawson, 1991; Guillon et al., 2014; Nakano et al., 2010; Riby & Hancock, 2009; von Hofsten et al., 2009). A recent meta-analysis of eye-tracking research on this topic concluded that autistic individuals spend less time attending to social stimuli (particularly faces and eyes) compared with their typically developing (TD) peers (Chita-Tegmark, 2016). This meta-analysis also revealed that differences in the stimuli used, particularly the complexity of the social content (e.g. the number of people in the scenes), account for contradictory findings in this area (Bar-Haim et al., 2006; Fletcher-Watson et al., 2008; Van Der Geest et al., 2002). It is important to note that the majority of these studies did not include a physically co-present social partner/stimuli and most were conducted on autistic children, making it hard to generalise these results to everyday, real-life social interactions in autistic adults (Tager-Flusberg, 1999). Schilbach et al. (2013) proposed that studying social cognition during ecologically valid social interactions will provide new insights into the cognitive mechanisms that underlie different psychiatric and developmental disorders that are characterised by disorders of social cognition. Arguably, the paucity of research on how autistic individuals navigate face-to-face social interactions has hindered progress in the field.

Only two studies that we are aware of have employed eye-tracking methods to investigate real-world social interactions among autistic individuals; one included autistic adults and the other autistic children (Freeth & Bugembe, 2019; Nadig et al., 2010). Freeth and Bugembe (2019) examined how the experimenter’s gaze direction (direct vs averted) influenced the allocation of social attention during conversation (speaking vs listening). They found that autistic adults were less likely to look at their social partner’s face than TD adults, especially when the partner’s gaze was directed at them. In line with previous studies, Freeth and Bugembe also found that TD adults made more fixations on their social partner’s eyes compared to their mouth, but this was not the case in autism (Corden et al., 2008). In contrast, Nadig et al. (2010) did not find any group difference in fixations to the experimenter’s face during a conversation, though a correlation analysis revealed that children who scored higher on the Autism Diagnostic Observation Schedule (ADOS) spent less time fixating on the experimenter’s face. The results of similar studies that have tested individual differences in the adult population are not entirely consistent with the evidence provided in these two studies. For example, the number of autistic traits self-reported by neurotypical adults did not correlate with the number of looks to the experimenter during a live interaction in Vabalas and Freeth (2016) or Freeth et al. (2013). However, a higher number of autistic traits manifested in a reduced tendency to visually explore the scene (Vabalas & Freeth, 2016), which the authors attributed to a local visual processing bias, as participants with a high number of autistic traits paid more attention to the details of specific areas in the scene and consequently explored the scenes less.

Studies that have examined eye movements during real-life interactions also suggest that social attention is modulated by topic of conversation in both autistic and non-autistic people. For example, Nadig et al. (2010) showed that both autistic and non-autistic individuals were more likely to look at their conversation partner’s face when talking about a topic of circumscribed interest than when talking about a general topic that they were not especially interested in. Similarly, Hutchins and Brien (2016) observed that during a Skype conversation, autistic children were less likely to look at the experimenter’s eyes when talking about emotions than when they were discussing general topics concerning occupations and lifestyles (TD children did not differ between topics). Perhaps discussing a familiar topic or a topic that does not involve emotion understanding (i.e. mentalising) with a social partner involves a lower processing cost than does discussion of an unfamiliar topic or a topic that requires emotion understanding. This is in line with numerous studies that have found higher levels of gaze aversion when individuals, regardless autism diagnosis, reply to questions that are difficult in nature (i.e. involve a high processing load; Doherty-Sneddon et al., 2002; Doherty-Sneddon & Phelps, 2005; Glenberg et al., 1998). This is important because social-communication skills in autistic people may be scaffolded when discussing topics that are familiar to them, or generally easy to process.

In the current study, we explored this by manipulating the perspective that speakers were prompted to adopt during conversation. We compared eye movements during a conversation between a participant and experimenter that either required the participant to mentalise about themselves, a person well-known to them, or a person who was unfamiliar to them. It has been shown that autistic individuals are particularly sensitive to the social interaction context. They make fewer references to their own beliefs, desires, or emotions in ambiguous and unstructured tasks compared to TD individuals (e.g. Bang et al., 2013), but show an intact ability to infer others’ perspectives and knowledge in tasks with a well-defined structure (Begeer et al., 2010). Interestingly, autistic adults, relative to TD adults, experience difficulty mentalising about a virtual character’s preferences when they are prompted to infer preferences for them (David et al., 2010). Previous research with neurotypical adults suggests that friends may have a better understanding of each others’ minds than strangers (e.g. Savitsky et al., 2011) and that the quality of social interaction is enhanced between pairs of friends vs strangers (Pollmann & Krahmer, 2018). Thus, we reasoned that discussions about the self and a familiar other might yield more typical patterns of eye gaze among autistic participants than discussions about an unfamiliar other, because self-relevant information is easier to process and structures cognition better than information relevant to others (especially unfamiliar others), among both TD people (Kuiper & Rogers, 1979; Sui & Humphreys, 2015; Symons & Johnson, 1997) and autistic people (e.g. Grainger et al., 2014; Lind et al., 2019; Williams et al., 2018).

It is clear that there is a lack of research comparing how mentalising during real-life social interactions influences eye gaze behaviour in autistic and TD individuals. In the current study, participants engaged in a semi-structured conversation with the experimenter, who prompted them with questions about themselves, someone familiar (such as their parent), or someone unfamiliar (a made-up character). These questions were designed to encourage participants to mentalise about different people. Participants’ answers and eye movements were recorded using a mobile eye-tracker and a voice recorder. The first aim was to examine group differences in the patterns of eye movements between autistic and TD adults during real-life social interactions, where the communication partner was physically present and language was unstructured. The second aim of this study was to establish for the first time the extent to which gaze to social and non-social aspects of the environment differ when autistic and TD participants are prompted to think about themselves, a familiar other person and an unfamiliar other person. This allows us to compare the processing costs of mentalising about the self and others in autism and helps us to gain a better understanding of self-referential processing in autism.

Previous eye-tracking research into social attention in autism suggests that autistic people attend significantly less to eyes/faces than TD people (Chita-Tegmark, 2016). Thus, in line with theories of atypical attention distribution in autism, we tested the prediction that autistic adults would be less likely to look at their conversation partner’s eyes/face, compared to TD adults. In addition, previous literature has shown that autistic people are more likely to look at their conversation partner’s face when discussing a topic that is easier for them to talk about (Hutchins & Brien, 2016; Nadig et al., 2010). Hence, we tested the prediction that participants in both groups would be more likely to fixate their partner’s face when talking about the self and familiar others (easier topic to talk about, thus reduced mentalising costs) compared to an unfamiliar other. Furthermore, we expected this effect to be even more pronounced in autistic people, due to the higher processing costs of mentalising about unfamiliar topics/people.

## Method

All methodological procedures were pre-registered on the Open Science Framework (OSF) website (see https://osf.io/g485j/).

### Participants

Initially, a total of 53 participants were recruited using the Autism Research at Kent (ARK) database. Participants on the database were recruited from a community sample in the areas of Kent, Essex and London in the United Kingdom, using a variety of recruitment strategies (e.g. newspaper adverts, contacting local groups, autism support groups and word-of-mouth). We deliberately avoided using university students to minimise differences in socioeconomic status between the groups. Three participants had to be excluded from the analysis due to technical problems (i.e. the experimenter could not obtain a successful calibration). Hence, the final sample consisted of 24 autistic and 26 TD participants, consistent with our pre-registered target sample size. These sample sizes were chosen a priori to be comparable or even exceed the sample sizes used in previous research that has examined eye movements during real-world interactions among autistic and TD participants (e.g. Hutchins & Brien, 2016; Nadig et al., 2010; Vabalas & Freeth, 2016) and our own previous work on pragmatic language comprehension in autistic adults (e.g. Barzy et al., 2020; Black et al., 2018, 2019; Ferguson et al., 2019). Post hoc calculations of power were conducted given the current sample size using the simr package in R (Green & MacLeod, 2016) and returned an estimated power of 87.9% with the significance level of α = .05 on 80% of occasions (as suggested by Cohen, 1988).

Participants in the two groups were matched on age, gender and IQ (measured by the Wechsler Abbreviated Scale of Intelligence (WASI); Wechsler, 1999; see Table 1 for demographic information). None of our participants had a diagnosis of dyslexia or reading comprehension impairments, and all were native speakers of English. All autistic participants had a formal diagnosis of Autistic Disorder, Asperger’s Syndrome or Pervasive Developmental Disorder Not-Otherwise Specified (*Diagnostic and Statistical Manual of Mental Disorders* (4th ed.; DSM-IV) or *Diagnostic and Statistical Manual of Mental Disorders* (5th ed.; DSM-5); American Psychiatric Association, 2013). Module 4 of the ADOS-2 (Lord et al., 2000) assessed the current autistic characteristics of autistic participants. ADOS assessments were conducted by a trained, research reliable researcher (see Table 1), and videos were double-coded to ensure reliability of scoring (inter-rater reliability was found to be excellent with intraclass correlation of .89). Twelve participants in the autistic group scored higher than 7 on the ADOS-2 Module 4 (i.e. the cut-off score, scores ranged between 1 and 21). All participants completed the Autism-spectrum Quotient (AQ; Baron-Cohen et al., 2001) as a self-report measure of autistic traits. Thirteen participants in the autistic group scored higher than 32 on AQ (i.e. the cut-off score), and scores ranged between 12 and 47. We retained all 24 autistic participants based on their formal clinical diagnosis, since ADOS and AQ measures are known to be relatively poor at predicting an autism diagnosis, particularly in adults (e.g. Ashwood et al., 2016; Risi et al., 2006).

**Table 1.** Demographic information (means and standard deviations) of participants in each group, where ***p < 0.001.

Demographics	Autistic	TD	*F*-value	*p*-value	$\eta_{p}^{2}$
	(*N* = 24)	(*N* = 26)			
Sex (m: f)	17:7	18:8	–	–	–
Age (years)	33.79 (11.14)	34.77 (17.40)	0.23	0.816	0.067
Verbal IQ	102.33 (11.23)	99.96 (9.31)	0.82	0.419	0.229
Procedural IQ	106.75 (20.24)	103.35 (11.51)	0.74	0.464	0.206
Full-scale IQ	104.71 (15.66)	102.00 (10.49)	0.72	0.473	0.204
Total AQ	31.29 (9.02)	19.31 (8.28)	4.90	<0.001***	1.383
ADOS2 Module4	8.00 (5.35)	–	–	–	–

TD: typically developing; AQ: Autism-spectrum Quotient; ADOS: Autism Diagnostic Observation Schedule.

### Materials and design

In order to establish an unfamiliar other, a short scenario was written by the experimenters (in two versions, describing a male or female character, matched to the participant’s gender). The scenario provided general information about the character (e.g. their occupation, where they are from, their hobbies; see Table 2 for the scenarios).

**Table 2.** Scenarios describing a female/male ‘unfamiliar’ character.

*Scenario describing a female character* Marina is from Rome in Italy. She is a 32-year-old chef, who loves cooking Italian food and baking cakes. She owns an Italian restaurant in London. Marina loves her family and likes to visit them in Rome regularly, especially on public holidays. She enjoys fashion and going shopping with friends. She also loves travelling in summer. For example, she really likes going to pretty little coastal towns in England where she can relax in the sun and read cookery books. She does not like rain at all so when the weather is wet, she tries her best to stay indoors. Marina also enjoys watching tennis and listening to classical music. She goes to see tennis matches or classical concerts in her free time. She has many Italian friends in London with whom she meets for a drink.	*Scenario describing a male character* Jack is from Rome in Italy. He is a 32-year-old chef, who loves cooking Italian food and baking cakes. He owns an Italian restaurant in London. Jack loves his family and likes to visit them in Rome regularly, especially on public holidays. He enjoys watching football on TV with friends. He also loves travelling in summer. For example, he really likes going to rustic little coastal towns in England where he can relax in the sun and read cookery books. He does not like rain at all so when the weather is wet, he tries his best to stay indoors. Jack also enjoys watching tennis and listening to classical music. He goes to see tennis matches or classical concerts in his free time. He has many Italian friends in London with whom he meets for a drink.

Nine questions, similar to those used in Vabalas and Freeth (2016), were designed to encourage conversation between the experimenter and the participant (see Appendix for the full set of questions). Participants were prompted to answer each question for themselves, for someone they know well (e.g. one of their parents or siblings), and for the unfamiliar character who was introduced in the scenario (e.g. ‘Tell me somewhere you/your mother/Marina would like to go over Christmas and why you think that?’). The questions were designed so that information in the scenario would provide some prompt to the unfamiliar other’s perspective, but participants would need to make further independent inferences about the character to elaborate with additional information (i.e. scenarios and questions were designed to encourage participants to mentalise about familiar and unfamiliar others). Questions were presented in the same order to all participants. Thus, the experiment employed a 3 × 2 mixed design, crossing the within-subjects variable Perspective (self/familiar other/unfamiliar other) with the between-subjects variable Group (ASD/TD).

To assess participants’ ToM abilities, we used the animations task (Abell et al., 2000). In this task, participants watched a series of four silent animation videos, in which two triangles interacted. Afterwards, participants were asked to describe the interactions between the triangles and say how they think the triangles felt at the end of each clip. To achieve the highest score, participants had to describe the triangles’ behaviour in terms of epistemic mental states, such as beliefs, intentions and deception. Participants’ audio responses were recorded for later transcription.

### Procedure

SensoMotoric Instruments (SMI) mobile eye-tracking glasses were used to record real-life eye movements. A front-facing camera on the glasses recorded a video of the scene (field of view: 60° horizontal, 46° vertical; resolution: 1280 × 960 pixels), as seen by participants, and binocular eye movements around this scene were recorded at a sample rate of 60 Hz (with 0.5° accuracy). Corrective lenses of the appropriate prescription could be attached to the eye-tracking glasses if necessary.

Participants were tested in a quiet laboratory at the School of Psychology, University of Kent. After giving consent to participate, participants were asked to read the unfamiliar other scenario, with the character matched to their gender. They were told that they would have a conversation with the experimenter about themselves, a familiar other (of their choosing, e.g. their mother) and an unfamiliar other (the character introduced in the scenario). Next, participants were fitted with the eye-tracking glasses, the experimenter ensured that they were comfortable and participants completed a three-point calibration and validation procedure. The experimenter sat in a chair opposite the participant, approximately 1 m away.

Participants were asked to choose a family member/friend that they could answer familiar other questions for and were reminded that they did not need to restrict their responses for unfamiliar other questions to the information provided in the scenario, but they should try to guess/expand their answers based on this information. The aim was to encourage participants to converse longer with the experimenter and to mentalise about the characters in the scenarios. Each participant responded to 27 questions in total (nine questions, in each of the three perspective conditions). Participants were encouraged to talk for approximately 30 s for each question. The experimenter prompted for further information when necessary and responded naturally to participants’ responses to facilitate the flow of conversation. The entire conversation task took approximately 20–30 min to complete. Finally, participants removed the eye-tracking glasses and completed the animations task on a computer. The whole experiment took approximately 40 minutes to complete.

## Results

All analysis procedures were pre-registered, and the full datasets and analysis scripts are available on the OSF web pages (see https://osf.io/g485j/).

### Animations task

To verify that ToM was diminished in our autistic sample, each verbal transcription was scored on a scale of 0–2 for accuracy (including reference to specific mental states), based on the criteria outlined in Abell et al. (2000). This resulted in a total score for each participant between 0 and 8 (with a higher score indicating better mentalising abilities). Twenty percent of transcripts were scored by two independent raters. Inter-rater reliability across all clips was excellent according to Cicchetti’s (1994) criteria (intraclass correlation = .85). Results showed that autistic participants were significantly impaired at describing the animations in terms of their mental states compared to TD participants (*M*s = 4.17 vs 5.38, respectively; *t*(48) = 2.04, *p* = 0.047, *d* = 0.57), which is in line with the means and effect sizes for autistic adults in previous studies (e.g. Brewer et al., 2017; Castelli et al., 2002).

### Eye movement data processing

**Figure 1.**
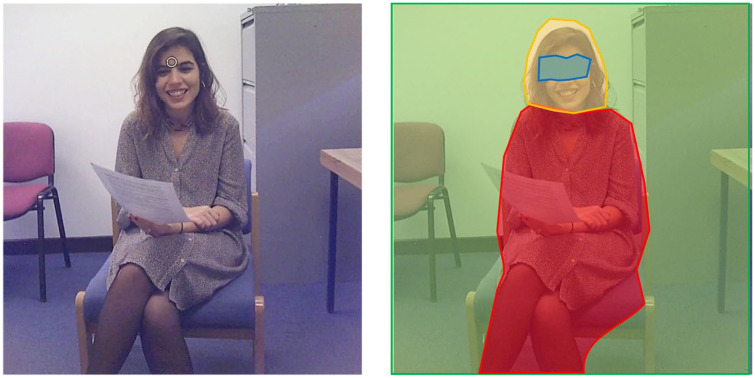
A screenshot of a typical view seen by participants during the experiment, and the corresponding AOIs for that view (eyes, face, body, background).

SMI BeGaze analysis software (3.7.59) was used to prepare fixation data for analysis. First, annotations were manually inserted into the timeline for each participant to indicate the onset and offset of each verbal response and to code for perspective (self, familiar other, unfamiliar other). Next, fixations during the verbal responses were assigned to one of the four areas of interest (AOIs): the experimenter’s eyes, face, body and background (see Figure 1). These AOIs were defined a priori based on previous research (Freeth & Bugembe, 2019; Vabalas & Freeth, 2016). We were particularly interested in fixations to the eyes and face (since studies have shown reduced attention to these areas in ASD) and the background (since studies have shown increased attention to this area while speaking). The background AOI was defined as any area in the scene except for experimenter. Analyses were conducted on the proportion of time spent fixating each AOI per condition and group, which was calculated separately for each participant and question (item) as summed duration of fixations on a specific AOI/sum of all fixation durations on all AOIs.

Linear mixed models and lmer in the lmer4 packages in Rstudio software were used to analyse the data (Bates et al., 2014; Version 1.1.453, R Core Team, 2016). Four separate models were used to analyse data from each of the four AOIs (eyes, face, body and background). Each model included fixed effects of Perspective and Group and random effects of items and participants. Since the effect of Group had two levels, it was contrast coded (−.5 vs .5). To accommodate the three levels of Perspective, deviation coded contrast schemes were used to compare each of the ‘other’ conditions to the baseline ‘self’ condition: Familiar other vs Self (Self (−.33), Familiar (.66), Unfamiliar (−.33)) and Unfamiliar other vs Self (Self (−.33), Familiar (−.33), Unfamiliar (.66)). Models also included the maximal random structure, including crossed random slopes for Group and Perspective within items and crossed random slopes for Perspective within participants. When the model did not converge, the random slopes that accounted for the least variance were removed (as suggested by Barr et al., 2013). Details of the final models used to analyse data for each AOI are presented in the supplementary materials. Note that due to space constraints, only significant effects are presented in the text. Full statistical effects are presented in Table 3.

**Table 3.** Model estimate, standard error (*SE*) and *t*/*z* value for each measure in each region, where *p < 0.05, **p < 0.01 and ***p < 0.001.

Regions	*Est.*	*SE*	*t*-value
Eyes			
Self vs FamiliarOther	−0.006	0.009	−0.589
Self vs UnfamiliarOther	−0.041	0.009	−4.269***
Group	0.072	0.054	1.32
Self vs FamiliarOther × Group	−0.023	0.019	−1.223
Self vs UnfamiliarOther × Group	0.006	0.019	0.331
Face			
Self vs FamiliarOther	−0.016	0.009	−1.793
Self vs UnfamiliarOther	−0.021	0.009	−2.428*
Group	0.134	0.052	2.557*
Self vs FamiliarOther × Group	0.011	0.017	0.627
Self vs UnfamiliarOther × Group	0.031	0.017	1.763
Body			
Self vs FamiliarOther	0.004	0.008	0.472
Self vs UnfamiliarOther	0.005	0.008	0.587
Group	−0.014	0.040	−0.349
Self vs FamiliarOther × Group	0.016	0.017	0.928
Self vs UnfamiliarOther × Group	0.027	0.017	1.621
Background			
Self vs FamiliarOther	0.012	0.012	1.066
Self vs UnfamiliarOther	0.045	0.012	3.849***
Group	−0.181	0.054	−3.354**
Self vs FamiliarOther × Group	0.006	0.023	0.263
Self vs UnfamiliarOther × Group	−0.050	0.023	−2.121*

### Eye movement analyses

#### Eyes

**Figure 2.**
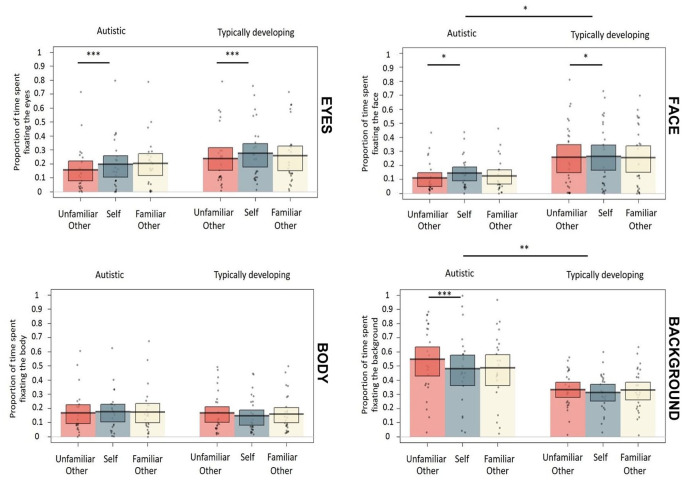
The proportion of time spent fixating each AOI in each condition and group (top left: eyes, top right: face, bottom left: body, bottom right: background). The plots show raw data points, a horizontal line reflecting the condition mean and a rectangle representing the Bayesian highest density interval. Asterisks indicate significant differences between conditions (*p < 0.05, **p < 0.01, ***p < 0.001).

Analysis revealed a significant effect of Perspective, with a greater proportion of fixation time spent on the experimenter’s eyes when participants were talking about the self than when they were talking about an unfamiliar other (*M* = 0.24 vs 0.20, Cohen’s *d* = 0.17). Neither the effect of Group nor the self versus familiar other Perspective contrast was significant, and Group did not interact with Perspective. Figure 2 shows the proportion of time spent fixating the experimenter’s eyes in each condition and group.

#### Face

**Figure 3.**
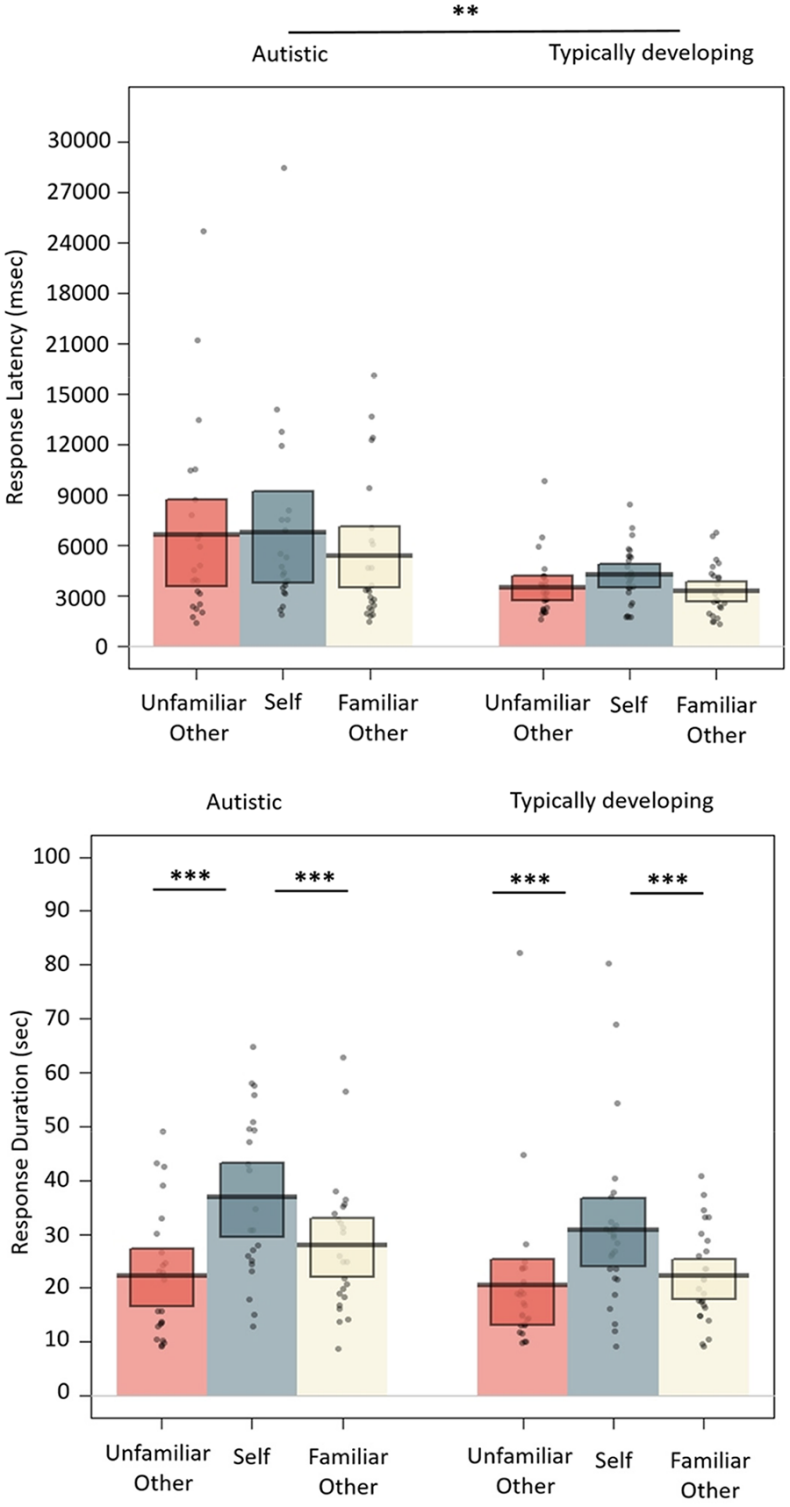
Response latency (in milliseconds) and response duration (in seconds) plotted by group and perspective condition. The plots show raw data points, a horizontal line reflecting the condition mean, and a rectangle representing the Bayesian highest-density interval. Asterisks indicate significant differences between conditions (**p < 0.01, ***p < 0.001).

Analysis revealed a significant effect of Group, reflecting the expected reduction in social attention among autistic participants; autistic adults in the current study spent significantly less time than TD comparison adults looking at the experimenter’s face (*M* = 0.13 vs 0.26, Cohen’s *d* = 0.59). A significant effect of Perspective showed that, overall, participants spent a greater proportion of time fixating on the experimenter’s face when they were talking about the self than when they were talking about an unfamiliar other (*M* = 0.21 vs 0.19, Cohen’s *d* = 0.10). Fixation patterns on the experimenter’s face did not differ between self and familiar other conversation perspectives, and Group did not interact with either Perspective contrast. Figure 3 shows the proportion of time spent fixating the experimenter’s face in each condition and group.

#### Body

None of the effects reached significance on this AOI.

#### Background

The effect of Group was significant, replicating previous research in showing that autistic adults spent a greater proportion of time fixating the background than the TD participants (*M* = 0.50 vs 0.33, Cohen’s *d* = 0.69). The effect of Perspective was also significant, reflecting a greater proportion of fixations on the background when participants were talking about an unfamiliar other compared to when they were talking about the self (*M* = 0.43 vs 0.39, Cohen’s *d* = 0.19). No difference in fixations to the background was found between self and familiar other conversation topics.

Moreover, Group significantly modulated the effect of self vs unfamiliar other Perspective (Cohen’s *d* = 0.19). To examine this effect further, post hoc tests compared fixations on the background for self versus unfamiliar other Perspective separately for each Group. In the autistic group, the effect of Perspective was significant (*Est.* = 0.069, *SE* = 0.019, *t* = 3.598, *p* < 0.001, Cohen’s *d* = 0.23), showing a greater proportion of fixations on the background when participants were talking about an unfamiliar other compared to when they were talking about the self (*M* = 0.54 vs 0.47). In the TD group, the effect of Perspective did not reach significance (*Est.* = 0.020, *SE* = 0.013, *t* = 1.480, *p* = 0.139, Cohen’s *d* = 0.10), thus fixation patterns around the background did not differ between self and unfamiliar other conversation topics among TD adults.

### Language production analyses

In addition to the main eye movement analyses, we conducted exploratory analyses (not included in the pre-registration) to compare the effects of Perspective and Group on features of language production, including the response latency and the response duration. Response latency was calculated as the time from the offset of the experimenter’s question to the onset of participants’ verbal response (in milliseconds). Response duration was calculated as the time from the onset of participants’ verbal response to the offset of their response (in seconds). Figure 3 shows the mean response latency and response duration in each condition and group.

Data for each measure was analysed using linear mixed models. Each model included fixed effects of Perspective and Group (coded as detailed above) and random effects of items and participants. Full statistical effects are presented in Table 4.

**Table 4.** Model estimate, standard error (*SE*), and *t/z* value for each response measure, where *p < 0.05, **p < 0.01, and ***p < 0.001.

Response Measures	*Est.*	*SE*	*t*-value
Response Latency			
Self vs FamiliarOther	−1085.19	595.79	−1.82
Self vs UnfamiliarOther	−428.78	851.26	−0.50
Group	−2551.28	910.38	−2.80**
Self vs FamiliarOther × Group	280.24	984.22	0.29
Self vs UnfamiliarOther × Group	−633.79	1247.00	−0.51
Response Duration			
Self vs FamiliarOther	−8.83	1.88	−4.70***
Self vs UnfamiliarOther	−12.20	1.88	−6.49***
Group	−4.40	3.26	−1.35
Self vs FamiliarOther × Group	0.29	3.76	0.08
Self vs UnfamiliarOther × Group	3.59	3.76	0.95

*Response Latency*: The effect of Group was significant, as autistic adults were slower to initiate their response to the experimenter’s questions compared to TD adults (*M* = 6271 vs 3707 ms, Cohen’s *d* = 0.57). Neither of the Perspective contrasts or the interactions between Group and Perspective were significant.

*Response Duration*: The effect of Perspective was significant, as participants talked longer about themselves compared to either a familiar or unfamiliar other (Self vs Familiar other *M* = 33.57 vs *M* = 24.83 s, Cohen’s *d* = 0.36; Self vs Unfamiliar other *M* = 33.57 vs 21.49 s, Cohen’s *d* = 0.49). Response durations were not influenced by Group, and Group did not modulate the effect of Perspective.

## Discussion

In a pre-registered experiment, we tested two novel objectives. First, we studied the pattern of eye movements during real-life social interactions in autistic and non-autistic individuals, a topic which has received little attention. Second, in the first study of its kind, we explored whether patterns of eye movements differ when individuals take the perspective of self versus others. Specifically, we were interested to see whether the pattern of gaze to social aspects of the environment (i.e. the experimenter here) differs when people (autistic or non-autistic) mentalise about the ‘self’ versus ‘someone they know’ (a familiar other) versus a ‘stranger’ (an unfamiliar other).

Between-group differences in eye movements revealed that autistic participants spent significantly *less* time than TD participants looking at the experimenter’s face regardless of the perspective adopted during conversation. Conversely, autistic individuals spent significantly *more* time than TD participants looking at the background when talking with the experimenter (particularly when the conversation was about an unfamiliar other). This was further supported by the results of our exploratory analysis on language production data, showing that in general autistic participants had longer response latencies, regardless of the perspective adopted. This could indicate that in general they found the conversation setting more cognitively demanding, which resulted in more gaze aversion.

These findings are largely in line with findings from Freeth and Bugembe (2019), the only eye-tracking study other than ours to examine social interaction in autistic adults with a physically present social partner. Similar to the current findings, Freeth and Bugembe found that, overall, autistic adults looked at the experimenter’s face for less time than their neurotypical peers, at least when the experimenter was directly looking at the participant (which is equivalent to our study, in which the experimenter always looked at the participant). However, our results contrast with the patterns seen among autistic children in Nadig et al. (2010), who did not differ from TD children in the proportion of time spent looking at their social partner’s face during conversation. We attribute these differences to methodological differences between studies. First, the sample size in Nadig et al. was relatively small (*N* = 12 ASD and N = 11 TD participants in their study compared to N = 24 and 26, respectively, in our study (*r* = 0.17 in Nadig’s study vs *d* = .59 in our study), meaning that it had particularly low statistical power to detect between-group differences. Second, Nadig et al’.s study tested children and involved an interaction with an adult. Currently little is known about the developmental trajectory of social attention in ASD, or how such processes might be affected by social dynamics between interlocutors (Gobel et al., 2015). In sum, there is a need to conduct cross-sectional and longitudinal research into gaze to social stimuli in real-life social interactions in autism.

One important finding in the current study was that significant between-group differences in the proportion of time spent looking at the eyes of the experimenter/social partner did not emerge. As noted in the introduction, there is a question about the extent to which gaze to eyes is impaired/diminished in ASD. While several studies have reported reduced gaze to eyes among autistic people (Chita-Tegmark, 2016; Corden et al., 2008; Guillon et al., 2014), other studies have failed to observe any such reduction (Bar-Haim et al., 2006; Fletcher-Watson et al., 2008; Van Der Geest et al., 2002). There is, arguably, a need for this issue to be addressed in further eye-tracking studies involving live, physically present social partners. The presence of live social partners adds ecological validity in studies of social attention, and in this way, the current study adds weight to the notion that gaze to eyes is not diminished among autistic adults during real-world interactions.

Regarding similarities between groups with respect to the perspective manipulation, we found that individuals in both groups were more likely to look at the experimenter’s eyes and face when talking about themselves compared to an unfamiliar other. In addition, our exploratory analysis on the production data showed that participants in both groups had longer response durations when talking about the self compared to either a familiar or an unfamiliar other, demonstrating that they found it easier to talk about themselves. This is in line with our prediction and shows that topic, specifically the adopted perspective in conversation can modulate social attention in both autistic and neurotypical adults. Arguably, these patterns reflect the differential costs of processing information/mentalising about oneself versus an unfamiliar other. These processing costs were magnified in the current study as discussions about the self and a familiar other relied on long-term memory and knowledge, whereas discussions about an unfamiliar other tapped shorter-term memories/knowledge provided in the character description at the beginning of the session. This is also consistent with previous studies, in which the topic of conversation modulated the social attention in both groups, with harder topics reducing the reciprocal interaction/engagement with the social partner (Nadig et al., 2010). Perhaps in this study self-relevant information was processed more easily and efficiently than information about others, especially unfamiliar others (see Lind et al., 2019). It may be that social attention is facilitated by the reduction in cognitive load associated with processing self-relevant versus other-relevant information. This idea is in keeping with findings that there is an increased cognitive load associated with representing the mental states of others when those mental states differ from one’s own compared to representing others mental states that are the same as one’s own (Apperly et al., 2008; Schneider et al., 2012). The fact, however, that attention to faces was reduced among participants with ASD even in the self- condition suggests that the advantage conferred by processing self-relevant over other-relevant information is not sufficient to overcome entirely the social attention difficulties observed in autistic people.

When it came to (non-social) eye gaze towards the background, we found a particularly important interaction between group and perspective. Autistic participants showed significantly increased gaze towards the background compared to TD participants across all three adopted perspectives in conversation, which likely reflects the social and cognitive load of managing the interaction (Doherty-Sneddon et al., 2013). However, the between-group difference in gaze to the background was reduced in the self-condition relative to the unfamiliar-other condition. This suggests that social attention was facilitated by self-reference among autistic participants, which is an important and novel finding. However, an alternative explanation for this particular finding is that autistic participants simply found it more difficult to recall details of, or construct details about, the unfamiliar other than they did to generate self-relevant information. While it is well-established autistic adults have difficulties with this kind of recall or construction process (e.g. Lind et al., 2014), we think it is unlikely that these difficulties affected patterns of eye-tracking in the current study. Notably, at the beginning of the experiment, the experimenter explicitly instructed the participant that the conversation task was not a memory test. Rather, the participant was encouraged to base his or her responses on what they think about the characters and not solely on what they read in the scenarios.

Several questions remain to be answered that are beyond the scope of this experiment. For example, it would be important to explore what the moderator cognitive mechanisms underlying this atypical visual attention are. Hutchins and Brien (2016) demonstrated that working memory abilities are correlated with number of fixations on the experimenter’s eyes (higher working memory scores resulted in more looks at the experimenter’s eyes). Further work needs to be carried out to establish whether executive functions modulate visual attention during real-life social interactions. Similarly, it would be interesting to examine whether effects on social attention differ according to formal diagnosis (i.e. autistic vs Asperger’s) or severity of autistic symptoms. In addition, previous studies have established that there are differences when looks to the mouth and eyes are coded separately, with autistic participants being more likely to look at the mouth of the experiments compared to their eyes (Chita-Tegmark, 2016). In this study, fixations on the mouth were aggregated with looks to other regions of the face, except for the eyes (as per our pre-registered analysis plan), so future research should separate these regions and explore this further. Furthermore, it is important to note that in this study, the questions were always asked in the same order – the experimenter always asked the self-related question first, then the familiar other question and finally the unfamiliar other question. Initiating the conversation by asking about self-related information may have facilitated responding to the familiar other questions. However, this would also imply that responses to the unfamiliar other condition would be facilitated by the two previous responses to familiar topics, and this effect was not apparent in the data here. It is possible then that difficulties with unfamiliar other processing are even greater without the scaffolding of a predictive flow of conversation.

In conclusion, this study explored the eye gaze behaviour during real-life social interactions, when autistic and non-autistic adults processed information about themselves, someone they know, or a stranger. Our results provide further evidence that social attention is atypical in ASD and that adults with this disorder show a pattern of eye gaze characterised by increased focus on non-social aspects of a scene at the expense of eye gaze towards (at least some) social aspects of the scene. Moreover, the current results add to evidence that the type of information being processed during conversation influences patterns of eye gaze/social attention. It is clear that social attention has a processing cost attached to it and this can be mitigated when the topic of conversation is relatively cognitively undemanding, i.e. relating to the self or a familiar other. This mitigation might enhance social attention in autistic people particularly. Further research into this question could be beneficial not only for our understanding of ASD but also for our understanding of the underlying basis of social attention more generally.

## Appendix

*Full set of experimental questions in each condition. Note that for each of the items in the following, conditions are listed in the order: Self, Familiar Other, and Unfamiliar Other.*

1a. Tell me somewhere you would like to go over Christmas and why you would like to go there?
1b. Tell me somewhere your mother/father would like to go over Christmas and why she or he would like to go there?
1c. where do you think Marina/Jack would like to go over Christmas and why you think Marina/John would like to go there?
2a. Tell me something you have to do during the week and something you like to do over the weekends?
2b. Tell me something your mother/father has to do during the week and something she or he likes to do over the weekends?
2c. What do you think Marina/Jack has to do during the week and what do you think she or he would like to do over the weekends?
3a. Could you tell me about a dish that you like to eat and whether you can cook it?
3b. Could you tell me about a dish your mother/father likes and whether she or he can cook it?
3c. Could you tell me about a dish that you think Marina/Jack likes and whether you think she or he can cook it?
4a. Tell me what kind of programmes you like to watch on TV and why do you like watching it?
4b. Tell me what kind of programmes your mother/father likes to watch on TV and why she or he likes watching it?
4b. Tell me what kind of programmes you think Maria/Jack would like to watch on TV and why you think she or he would like watching it.
5a. Tell me one thing you like about living in England and one thing you don’t like about living in England?
5b. Tell me one thing your mother/father likes about living in England and one thing she or he doesn’t like about living in England?
5c. Tell me one thing you think Marina/Jack likes about living in England and one thing you think she or he doesn’t like about living in England?
6a. Name a place you would like to visit in England and why would you like to visit there?
6b. Name a place your mother/father would like to visit in England and why she or he would like to visit there?
6c. Name a place you think Marina/Jack would like to visit in England and why you think they would like to visit there?
7a. Tell me what you like to buy when you go shopping and why you like to buy it?
7b. Tell me what your mother/father likes to buy when she or he goes shopping and why she or he likes to buy it?
7c. Tell me what do you think Marina/Jack would like to buy when she or he goes shopping and why you think she or he would like to buy it?
8a. Tell me something you like to do in your spare time, like a sport or an activity, then describe some of the rules of this sport/activity?
8b. Tell me something your mother/father likes to do in her or his spare time, like a sport or an activity, then describe some of the rules of this sport/activity?
8c. Tell me something you think Marina/Jack likes to do in her or his spare time, like a sport or an activity, then describe some of the rules of this sport/activity?
9a. Tell me who is your favourite celebrity and why you like him or her?
9b. Tell me who is your mother/father’s favourite celebrity and why she or he likes him or her?
9c. Tell me who you think is Marina’s/Jack’s favourite celebrity and why you think Marina/Jack would like him or her?

## Notes

1. We acknowledge recent debates about the terminology used to describe autism and in this study adopt the identity-first language recommended by autistic adults and parents in Kenny et al. (2016).
